# Mono-ADP-ribosylation sites of human CD73 inhibit its adenosine-generating enzymatic activity

**DOI:** 10.1007/s11302-021-09832-4

**Published:** 2021-12-27

**Authors:** Julia Hesse, Mona K. Rosse, Bodo Steckel, Bernhard Blank-Landeshammer, Svenja Idel, Yvonne Reinders, Albert Sickmann, Norbert Sträter, Jürgen Schrader

**Affiliations:** 1 Department of Molecular Cardiology, Medical Faculty and University Hospital Düsseldorf, Heinrich-Heine-University Düsseldorf, Universitätsstrasse 1, 40225 Düsseldorf, Germany; 2 Leibniz-Institut für Analytische Wissenschaften-ISAS-e.V., Dortmund, Germany; 3 Institute of Bioanalytical Chemistry, Center for Biotechnology and Biomedicine, Leipzig University, Leipzig, Germany

**Keywords:** Mono-ADP-ribosylation, Post-translational modification, NT5e, CD296, Adenosine

## Abstract

CD73-derived adenosine plays a major role in damage-induced tissue responses by inhibiting inflammation. Damage-associated stimuli, such as hypoxia and mechanical stress, induce the cellular release of ATP and NAD^+^ and upregulate the expression of the nucleotide-degrading purinergic ectoenzyme cascade, including adenosine-generating CD73. Extracellular NAD^+^ also serves as substrate for mono-ADP-ribosylation of cell surface proteins, which in human cells is mediated by ecto-ADP-ribosyltransferase 1 (ARTC1). Here we explored, whether human CD73 enzymatic activity is regulated by mono-ADP-ribosylation, using recombinant human CD73 in the presence of ARTC1 with etheno-labelled NAD^+^ as substrate. Multi-colour immunoblotting with an anti-etheno-adenosine antibody showed ARTC1-mediated transfer of ADP-ribose together with the etheno label to CD73. HPLC analysis of the enzymatic activity of in vitro-ribosylated CD73 revealed strong inhibition of adenosine generation in comparison to non-ribosylated CD73. Mass spectrometry of in vitro*-*ribosylated CD73 identified six ribosylation sites. 3D model analysis indicated that three of them (R328, R354, R545) can interfere with CD73 enzymatic activity. Our study identifies human CD73 as target for ARTC1-mediated mono-ADP-ribosylation, which can profoundly modulate its adenosine-generating activity. Thus, in settings with enhanced release of NAD^+^ as substrate for ARTC1, assessment of CD73 protein expression in human tissues may not be predictive of adenosine formation resulting in anti-inflammatory activity.

## Introduction

**Fig. 1:**
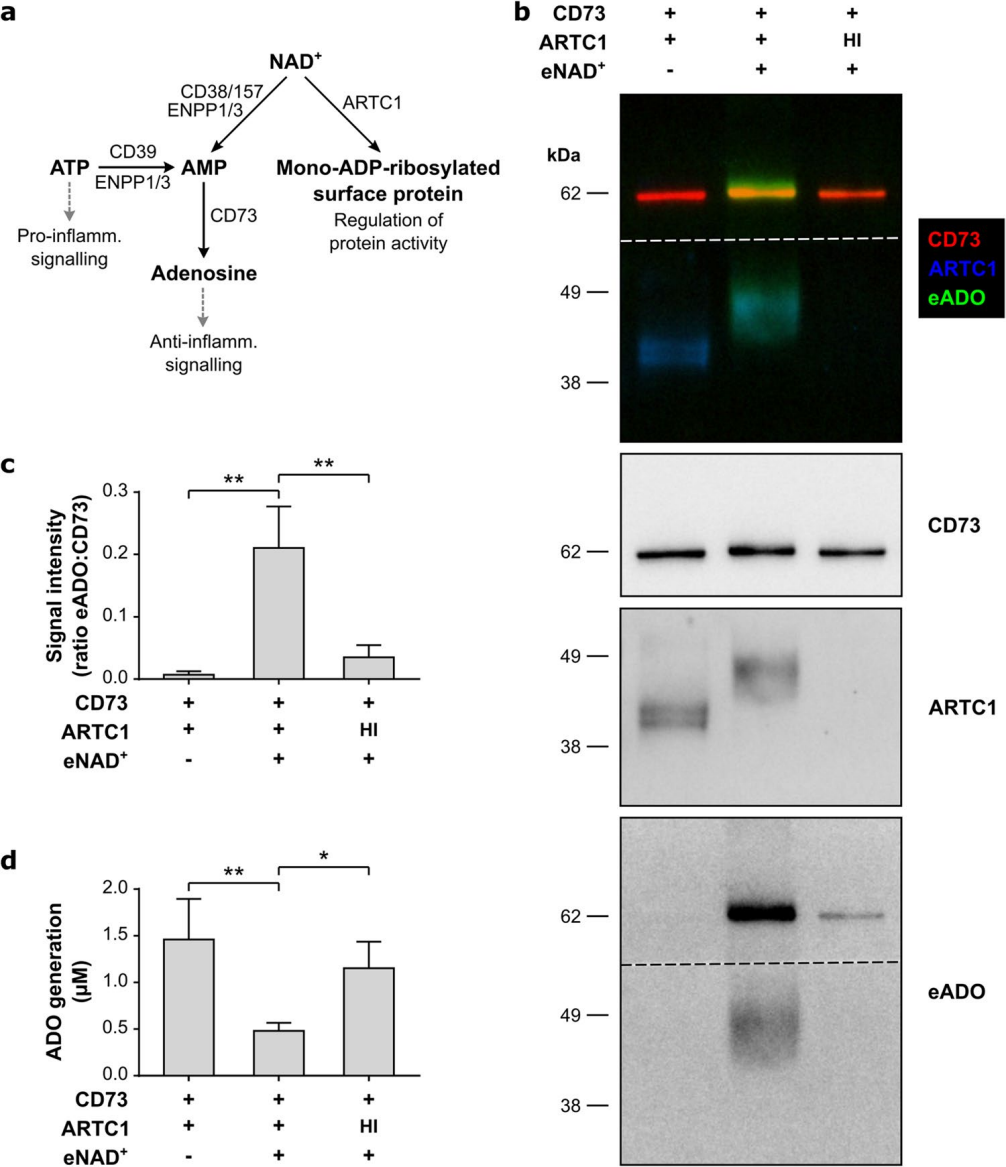
Ribosylation of CD73 by ARTC1. **a** Scheme of extracellular nucleotide metabolism. **b–c** Human recombinant CD73 was incubated with ARTC1 in the presence of 320 µM etheno-labelled NAD^+^ (eNAD^+^) for 16 h at 30 °C. Transfer of etheno label from eNAD^+^ on CD73 and ARTC1 was detected by multi-colour immunoblotting using etheno-adenosine (eADO)-, CD73-, and ARTC1-specific primary antibodies in combination with fluorochrome-labelled secondary antibodies. Representative blots are shown in **b**. In the top panel, an overlay of CD73, ARTC1, and eADO signals is displayed. Dashed lines indicate where the blot membrane was cut to allow separate antibody incubation (upper part, α-eADO with α-CD73; lower part, α-eADO with α-ARTC1). eADO signals co-localized with CD73 were quantified and normalized to CD73 signals as shown in **c**. Means ± SD (*n* = 4 independent experiments). **d** Human recombinant CD73 was ribosylated as described in (**a**) and analysed for enzymatic activity by HPLC. Generation of ADO was quantified after 5 min of incubation with 20 µM AMP. Means ± SD (*n* = 4 independent experiments). One-way ANOVA and Tukey’s multiple comparisons test. *P < 0.05, **P < 0.01. *HI* heat-inactivated ARTC1

The release of nucleotides in the extracellular space by lytic and nonlytic mechanisms is a major cellular response to tissue damage. Both ATP and NAD^+^ act as ‘danger signals’ and trigger inflammation [1]. Extracellular NAD^+^ can be metabolized to adenosine by an ecto-enzyme cascade [2] or serve as substrate for mono-ADP-ribosylation of cell surface proteins [3] (Fig. 1a).

Ecto-5’-nucleotidase CD73, converting AMP to adenosine, is a central member of the ATP- and NAD^+^-degrading cell surface enzyme cascades, controlling the levels of free anti-inflammatory adenosine that shields the organism from excessive inflammatory responses [4]. In the context of cancer, its immunosuppressive activity may also be detrimental, and this is presently exploited in cancer immunotherapy using inhibitors of CD73 [5].

Several mechanisms are known to control CD73 expression and enzymatic activity, including induction of transcription by hypoxia [6], competitive inhibition by micromolar concentrations of ATP and ADP [2], modulation of enzymatic activity by binding to extracellular matrix proteins [7], and attenuation of activity by post-translational modification via N-glycosylation [8].

Arginine-specific mono-ADP-ribosylation mediated by ecto-ADP-ribosyltransferases (ARTCs) has been established as important regulatory post-translational modification of various cell surface proteins [3]. Mono-ADP-ribosylation attaches a bulky (~ 540 Da), negatively charged ADP-ribose moiety to the target protein that can modify protein function, e.g. by sterically blocking interaction sites or modulating protein conformation [3]. While in mice there are three GPI-anchored ARTC family members (ARTC1, ARTC2.1, ARTC2.2), mono-ADP-ribosylation at the surface of human cells is exclusively dependent on ARTC1 [9]. A recent proteomic analysis identified hundreds of ARTC1 targets in mouse skeletal and heart muscle tissue, associated with signal transduction, transmembrane transport, and muscle function [10].

Since mono-ADP-ribosylation and NAD^+^-dependent adenosine generation compete for NAD^+^ at the cell surface, we explored, whether human CD73 and its adenosine-generating activity is a target of ARTC1.

## Methods

### In vitro ribosylation of recombinant human CD73

Recombinant human CD73 (150 ng; produced in CHO cells, #5795-EN, R&D Systems) was incubated with recombinant cynomolgus ARTC1 (60 ng; produced in HEK 293 cells, #LS-G49947, LSBio) and etheno-NAD^+^ (eNAD^+^, 320 µM; Biolog Life Science Institute) as substrate in potassium phosphate buffer (50 mM, pH 7.5) [11] in a total volume of 13 µl for 16 h at 30 °C. This recombinant cynomolgus ARTC1 protein preparation with 95.1% identity (BLAST) to human ARTC1 was chosen for this study, since commercially available recombinant human ARTC1 proteins are generated in yeast or *E. coli*, expression systems that produce proteins with non-mammalian glycosylation profiles that potentially can impair activity. For non-ribosylated controls, ARTC1 was either not added to the reaction or was heat-inactivated for 10 min at 95 °C prior to incubation.

### Multi-colour immunoblotting

Immunoblotting was performed using the Bolt Bis–Tris Mini Gel system and the iBlot2 Western Blotting system (Thermo Fisher Scientific). Seven microlitre of the ribosylation reaction was supplemented with 2.5 µl Bolt LDS Sample buffer and 1 µl Bolt Sample Reducing Agent. After incubation for 10 min at 70 °C, gel electrophoresis and protein transfer on PVDF membranes were performed according to the manufacturer’s instructions. Membranes were incubated with rabbit-anti-CD73 (1:1,000, clone D7F9A, #13,160, Cell Signaling Technology), rabbit-anti-ARTC1 (1:100, #ab71295, Abcam), and mouse-anti-etheno-adenosine (1:400, clone 1G4, #MA1-16,884, Thermo Fisher Scientific) primary antibodies and Alexa Fluor (AF) Plus 488-anti-rabbit, AF Plus 555-anti-rabbit, and AF Plus 647-anti-mouse secondary antibodies (Thermo Fisher Scientific). To allow detection of CD73 and ARTC1 on the same plot, membranes were cut in-between protein ladder bands 62 kDa and 49 kDa (SeeBlue Plus2, Thermo Fisher Scientific) and the pieces were separately incubated. Signals were detected with an iBright FL1000 Imaging System (Thermo Fisher Scientific). As measure for the ribosylation level, intensities of eADO signals that were co-localized with CD73 bands were quantified and normalized to CD73 signals.

### CD73 activity assay

To assess CD73 enzymatic activity, 2 µl of the ribosylation reaction was transferred into 798 µl of 20 µM AMP and incubated for 5 min at 37 °C. The enzymatic reaction was terminated by addition of 80 µl of 10% 5-sulfosalicylic acid. After centrifugation at maximal speed for 10 min at 4 °C, the supernatant was subjected to high performance liquid chromatography (HPLC) analysis with an ACQUITY UPLC H-Class System equipped with a CORTECS C18 UPLC column (3.0 × 150 mm, particle size 1.6 µm) (Waters). Purine separation was performed as previously described [12], using a linear gradient of buffer A (200 mM KH_2_PO_4_/200 mM KCl, pH 6) and buffer B (200 mM KH_2_PO_4_/200 mM KCl/7.5% acetonitrile, pH 6). Absorbance was measured at 254 nm. For each sample, technical triplicates of the CD73 activity assay were analysed.

### Mass spectrometry

To generate 1 µg of ribosylated recombinant human CD73 for mass spectrometric analysis, the ribosylation reaction described above was up-scaled, and non-labelled NAD^+^ was used as substrate for ARTC1. For non-ribosylated CD73 as control, the incubation was performed without NAD^+^.

Disulfide bonds were reduced at 56 °C for 30 min by addition of 10 mM dithiothreitol (DTT, Sigma Aldrich), followed by alkylation with 30 mM Iodoacetamide (IAA, Roche) at ambient temperature for 30 min. After ethanol precipitation for 1 h at − 40 °C, protein digestion was performed overnight at 37 °C using trypsin (Promega) at a ratio of 1:20 (w/w) to the protein concentration in digestion buffer (50 mM ammonium bicarbonate (Sigma Aldrich); pH 7.8; 0.2 M GuHCl (Sigma Aldrich)). Digests were quenched using trifluoroacetic acid (TFA, Sigma Aldrich; final concentration 1%) before adjusting concentration to 3 pmol.

The HPLC instrument was an UltiMate 3000 Nano LC system from Dionex, and the mass spectrometer was an Orbitrap Fusion Lumos from Thermo Scientific equipped with a nano-electrospray ion source. Tryptic peptides were separated using nano-reversed phase-HPLC on a 75 µm × 25 cm PepMap RSLC from Thermo Scientific using a 1-h gradient of 3–42% acetonitrile in 0.1% formic acid at a flow rate of 300 nl/min. The MS1 survey scans of the eluting peptides were executed with a resolution of 120,000, recording a window between 300.0 and 1,500.0 m/z. MS2 scans were executed with a resolution of 30,000 with either high-energy collision-induced dissociation (HCD) or electron-transfer/higher-energy collision dissociation (EThcD) for fragmentation. The normalized collision energy (nCE) was set at 32.0% for HCD and 30% for the respective EThcD. Data evaluation was performed with PEAKS 7.5 and Progenesis QI (Waters) using 10 ppm for precursor mass tolerance, 0.02 Da for fragment mass tolerance, tryptic digest, a maximum of 3 missed cleavages, fixed (C [+ 57.002146 Da]) and variable (M [+ 15.99491 Da], ADP-Ribosylation (R) [+ 541.061110 Da) modification, and FDR of 1% on peptide level.

‘Estimated modification rate’ was calculated based on the difference of average normalized abundance of non-modified peptides for a given modification site.

### 3D model analysis of CD73

For the analysis of the structural environment of the ribosylated arginines of CD73, dimer structures in the open (PDB id 4h2g) and closed (4h2i) states were generated in COOT [13]. The proteins were superimposed based on the two C-terminal domains in PYMOL (www.pymol.org). The environment of the arginine residues was inspected in the open and closed states and in a morph between the two states generated by PYMOL.

### Statistics

Experimental replicates were generated using independent ribosylation reactions. Values are presented as means ± SD. Statistical analyses of immunoblotting and CD73 enzymatic activity assays using one-way ANOVA with Tukey’s multiple comparisons test were performed with GraphPad Prism. The threshold for statistical significance was set at *P* < 0.05.

## Results

To assess whether human CD73 is a target of ARTC1-mediated mono-ADP-ribosylation, the ribosylation reaction of recombinant human CD73 in the presence of recombinant cynomolgus ARTC1 and etheno-labelled NAD^+^ (eNAD^+^) was analysed in vitro (16 h at 30 °C). As visualized by immunoblotting, ARTC1 mediated the transfer of etheno-adenosine (eADO)-containing ADP-ribose moieties from eNAD^+^ onto CD73 (Fig. 1b,c). This transfer led to a shift in molecular weight between non-ribosylated CD73 (61.1 ± 0.5 kDa) and ribosylated CD73 (62.8 ± 1.2 kDa), corresponding to three ADP-ribose moieties on the average. Heat inactivation of ARTC1 abolished the transfer of the etheno-label (Fig. 1b,c). ADP-ribose moieties were also transferred onto ARTC1 itself (Fig. 1b) and changed the molecular weight from 41.1 ± 0.5 to 46.7 ± 0.3 kDa, corresponding to 10 ADP-ribose moieties. Auto-ribosylation of ARTC1 has been reported before [14].

To analyse the functional impact of ARTC1-mediated mono-ADP-ribosylation, the enzymatic activity of CD73 was quantified by HPLC. As shown in Fig. 1d, the capacity of ribosylated CD73 to form adenosine was severely impaired. Again, heat inactivation prevented this effect.

**Fig. 2:**
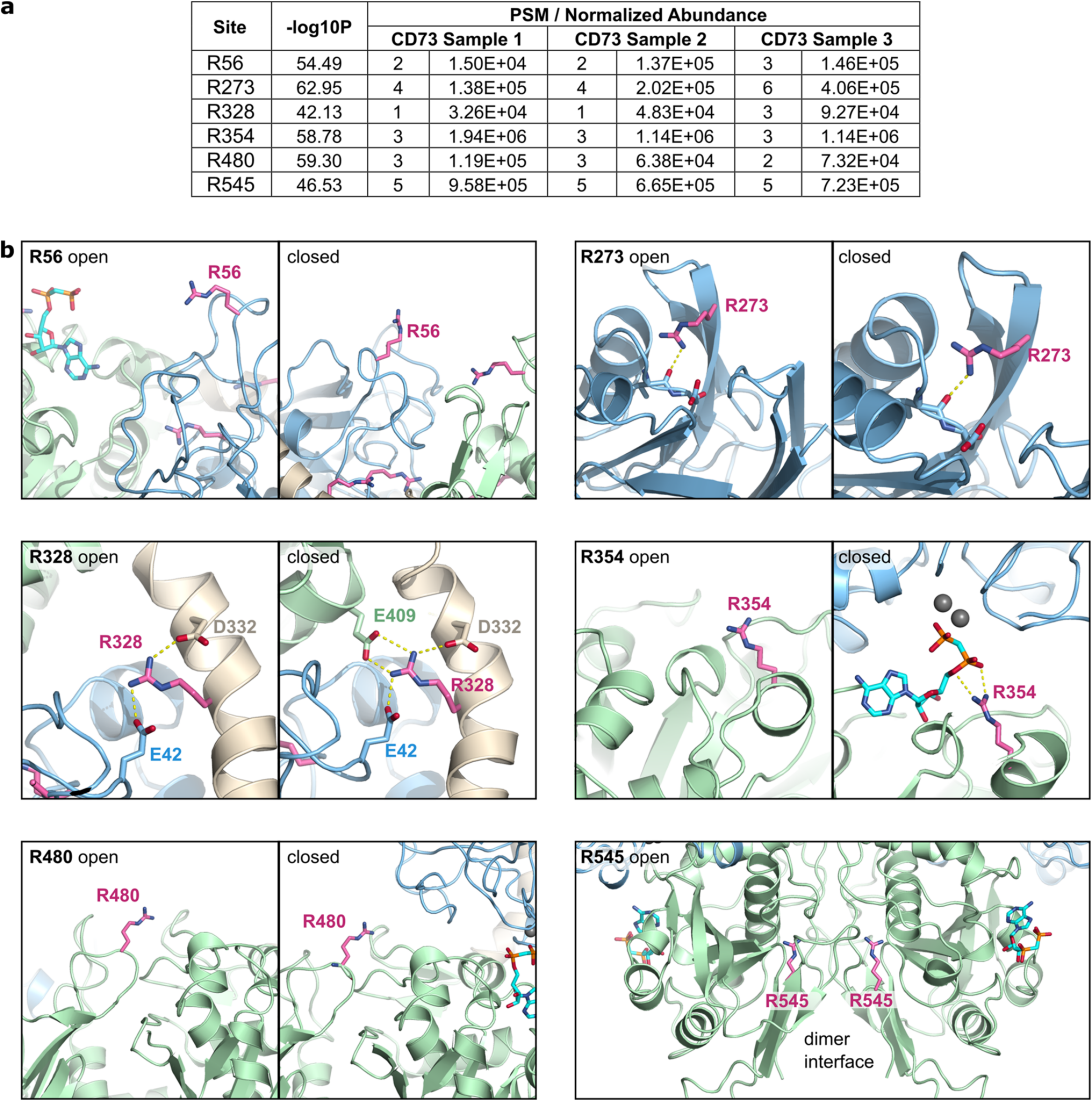
Sites of ARTC1-mediated ribosylation in CD73. **a** Human recombinant CD73 was incubated with ARTC1 in the presence of 320 µM etheno-labelled NAD^+^ for 16 h at 30 °C. Ribosylated arginines in CD73 were identified by mass spectrometry (*n* = 3 independent ribosylation reactions). Only sites observed in all three analysed ribosylated CD73 samples are shown. ADP-ribosylation sites, negative logarithmic *p*-values, peptide spectrum matches (PSM), and the calculated normalized abundances are given. **b** Environment of the identified arginines in open and closed CD73 conformations

For identification of the mono-ADP-ribosylation sites in CD73, mass spectrometry of ribosylated CD73 was performed. As summarized in Fig. 2a, six ribosylated arginines were identified in all three experimental replicates. These ribosylation sites were located at different positions throughout the CD73 protein sequence.

Using available crystal structures of the activity-associated conformational switch of the dimeric CD73 between open and closed states [15], the accessibility of the identified arginines and the potential impact of mono-ADP-ribosylation at these sites on CD73 enzymatic activity was analysed. As shown in Fig. 2b, R56 is exposed in the open and closed CD73 states. Since this is also true for all conformations created in the morph, its ADP-ribosylation is unlikely to affect catalysis. R273 is located at the opposite site of the domain interface. It is hydrogen bonded to the carbonyl group of V236 in both conformations. Upon ribosylation the side chain may reorient to the solvent. Its ribosylation is unlikely to affect the domain movement but may destabilize the fold of the N-terminal domain to a small extent. R328 is located in the helix linking the two CD73 domains. Its side chain interacts with E409 in the closed conformation via a salt bridge. After ribosylation, R328 needs to adopt another side chain conformation such that the closed state can form. This will result in a loss of the R328-E409 salt bridge and may result in a destabilization of the closed state. R328 also forms salt bridges with E42 and D332. The loss of these interactions may destabilize the interface between the C-domain and the domain-linking helix. Therefore, it is conceivable that mono-ADP-ribosylation of R328 influences catalysis. R354 is partially buried by the C-terminal domain in the open state, but the guanidium group is accessible. In the closed state, the side chain is not accessible for enzymatic modification due to the presence of the N-terminal domain. R354 is probably a crucial residue for catalysis as it is involved in substrate binding at the active site by interacting with the phosphate groups of the nucleotide substrate in the closed state. Mono-ADP-ribosylation of R354 likely inactivates CD73 completely. R480 is located close to the dimer interface. It is solvent exposed in both conformations, and its ribosylation is likely not influencing enzyme function. R545 is buried at the dimer interface and involved in a salt bridge with D366. It appears to be hardly accessible for ribosylation, unless this region close to the C-terminus and the GPI anchor can adopt alternative orientations, possibly involving dimer dissociation. In the ribosylated state, R545 would likely prevent dimer formation.

## Discussion

This study reveals post-translational modification sites for ARTC1-mediated mono-ADP-ribosylation in human CD73 that severely impairs its adenosine-generating enzymatic activity. We identified six ribosylation sites in in vitro ribosylated CD73, of which R328, R354, and R545 seem to have the potential to interfere with CD73 enzymatic activity.

In contrast to N-glycosylation, which was recently reported to attenuate CD73 activity in human hepatocellular carcinoma [8], ARTC1-mediated mono-ADP-ribosylation takes place at the cell surface and depends on the availability of NAD^+^ in the extracellular microenvironment. Our results suggest that in settings with pronounced nucleotide release, such as tissue damage and inflammation, ARTC1-mediated ribosylation of CD73 can severely impair adenosine generation and signalling. This mechanism might switch the balance of an adenosine-dominated anti-inflammatory environment towards sustained ATP-mediated pro-inflammatory signalling. A role for ARTC1-mediated mono-ADP-ribosylation in inflammatory cytokine release has already been proposed in LPS-stimulated human alveolar epithelial cells [16]; however, the target proteins of mono-ADP-ribosylation were not identified. Since human alveolar epithelial cells highly express CD73 [17], an ARTC1-mediated inactivation of CD73 might have been involved. Very recently, we reported profound inhibition of CD73 activity on B cells from patients with systemic lupus erythematosus (SLE) [18], which most likely was due to a yet unidentified post-translational modification of CD73. In view of the present findings, it is tempting to speculate that ARTC1-mediated CD73 ribosylation might be the underlying mechanism for CD73 silencing in SLE.

Ribosylation-mediated CD73 inactivation may potentially be reversed by extracellular phosphodiesterase activity as has been reported for mono-ADP-ribosylated α7 integrin on skeletal muscle cells [19]. In this mechanism, the AMP bulk is cleaved from ADP-ribose, possibly releasing steric interference [3]. This leaves a phosphoribose moiety at the site, which blocks re-ADP-ribosylation. Interestingly, the ectoenzyme ENPP1, which also displays NAD^+^-degrading activity, has been recently identified to mediate this processing in vitro [20]. Thus, ENPP1 might serve as a dual negative regulator of ARTC1 activity at the cell surface by competing for NAD^+^ as substrate and releasing steric interference. It remains to be investigated, whether ENPP1 can counteract ARTC1-mediated CD73 inactivation and restore adenosine generation.

While this study used soluble recombinant proteins, CD73 and ARTC1 at the cell surface in vivo are tethered to the plasma membrane by GPI-anchors. Theoretically this may restrict their potential interaction, and the individual ribosylation sites identified in this study may be less relevant. These issues need to be addressed in future studies. On the other hand, it is well known that GPI-anchors facilitate the enrichment in specialized membrane microdomains, lipid rafts, which enhances the likeliness of direct interactions of CD73 and ARTC1 at the cell surface. Very recently, Leutert et al. found that the mouse ARTC family member ARTC2.2 mediated ribosylation of CD73 at R149 on mouse T cells and that deletion or inhibition of ARTC2.2 on mouse CD8^+^ T cells resulted in an enhanced adenosine-generating activity in the presence of NAD^+^ [21]. In humans, ARTC2 is not expressed as functional protein [22], and there are also profound differences in the physiological role of CD73 between mice and men [23].

ARTC1 and CD73 may also interact as naturally occurring soluble forms in vivo, e.g. at sites of inflammation. ARTC2.2 has been shown to be shed from mouse T cells in response to activation [24], and this soluble form of ARTC2.2 can ribosylate cytokines, thereby regulating their signalling function [25]. The existence of non-cell bound human CD73 is also well documented, e.g. the release after hydrolysis of the GPI anchor by phospholipases [26] or CD73-mediated adenosine production by T cell-derived extracellular vesicles [27].

Future studies will be necessary to explore the functional consequences of ARTC1-mediated CD73 mono-ADP-ribosylation in the human system, such as its potential impact on immune cell regulation in physiological and pathophysiological settings.

## Data Availability

Data are available upon request to the corresponding author.
